# Can anti-erosion dentifrices also provide effective plaque control?

**DOI:** 10.1111/j.1601-5037.2010.00480.x

**Published:** 2011-08

**Authors:** PG Bellamy, M Prendergast, R Strand, Z Yu, TN Day, ML Barker, AJ Mussett

**Affiliations:** Procter & Gamble, London Innovation CentreEgham, Surrey, UK; Procter & Gamble, Beijing Technical CentreBeijing, PR China; Procter & Gamble, Mason Business CenterMason, OH, USA

**Keywords:** clinical trial, dental hygiene, dentifrice/gel, fluoride, oral hygiene, plaque control, plaque formation

## Abstract

**Objective:**

While gingivitis and caries continue to be prevalent issues, there is growing concern about dental erosion induced by dietary acids. An oral hygiene product that protects against all these conditions would be beneficial. This study investigated the potential of two anti-erosion dentifrices to inhibit plaque.

**Methods:**

This was a randomized, three-period, two-treatment, double-blind, crossover study evaluating a stannous chloride/sodium fluoride dentifrice (SnCl_2_/NaF, blend-a-med® Pro Expert) and a popular anti-erosion dentifrice (NaF, Sensodyne® ProNamel™). During Period 3, subjects were randomized to repeat one treatment to evaluate any product carryover effects. Each treatment period was 17 days. Test dentifrices were used with a standard manual toothbrush. Digital plaque image analysis (DPIA) was employed at the end of each period to evaluate plaque levels (i) overnight (am prebrush); (ii) post-brushing with the test product (am post-brush); and (iii) mid-afternoon (pm). Analysis was conducted via an objective computer algorithm, which calculated total area of visible plaque.

**Results:**

Twenty-seven subjects completed the study. At all time points, subjects had statistically significantly (*P* ≤ 0.0001) lower plaque levels after using the SnCl_2_/NaF dentifrice than the NaF dentifrice. The antiplaque benefit for the SnCl_2_/NaF dentifrice versus the NaF dentifrice was: am prebrush = 26.0%; am post-brushing = 27.9%; pm = 25.7%.

**Conclusions:**

The SnCl_2_/NaF dentifrice provided significantly greater daytime and overnight plaque inhibition than the NaF toothpaste. When recommending dentifrice to patients susceptible to dental erosion, clinicians can consider one that also inhibits plaque.

## Introduction

Dental erosion is defined as the loss of tooth substance by acid exposure not involving bacteria (1). The oral health problem of dietary induced dental erosion is not a new one. There have been reports in the literature for decades documenting the problems associated with erosion of the hard tissues due to dietary acids (2, 3). However, more recently, epidemiological studies and case reports have indicated that dental erosion is a growing problem, particularly in the last 10–20 years (4, 5). Periodontal diseases and caries continue to be prevalent issues in oral medicine. But indeed, with caries amongst children in developed countries at historically low levels (6), erosion is now becoming a focus of dental research because it has the potential to cause significant dental health problems as the current child population ages (4).

To combat this growing risk to hard tissue health, manufacturers of oral care products have begun to market daily use products with claimed erosion protection benefits. There are a number of technological routes available, providing varying levels of protection against dietary acids, which can cause dental erosion. One popular approach is the use of a common caries-preventive fluoride as a protective system against dietary erosion. Such fluoride sources, like sodium fluoride (NaF), have been reported to provide some level of protection against erosive loss of hard tissue (7, 8). Conversely, there are also reports of research where fluoride has provided no, or very limited, protective benefit (9, 10). Recent increases in observed levels of erosion (4) suggest that the efficacy of regular fluoride toothpastes alone as preventive measure against dental erosion is not sufficient.

A second common route to erosion prevention found within the literature is the use of stannous compounds and in particular stannous fluoride (SnF_2_) (11, 12). The mode of action for SnF_2_ is still not completely understood and continues to be an area of current research (13). The formation on the hard tissue of a micro-thin tin fluorophosphate layer has been suggested (14), while other research suggests incorporation of stannous/tin into the first few microns of enamel is essential for anti-erosion efficacy (13). This has led to discussion of the relative roles of stannous ions (Sn^2+^) and fluoride ions (F^−^) (12) with recently reported *in vitro* work (15) indicating that in isolation each is only moderately effective, but when in combination, they are highly effective.

While daily use products to help prevent dental erosion is clearly a growing need, other oral health issues remain a significant problem. Poor gingival health has not surprisingly been reported by periodontal specialists to negatively impact the quality of life of patients (16). There remains a considerable need for daily use products, which will help prevent plaque-associated conditions such as gingivitis and dental caries. Effective plaque control is the aspect of oral care which is mostly under the control of the patient, and includes use of mechanical hygiene tools to remove plaque and effective chemotherapeutic products, which help inhibit plaque regrowth (17).

The need to address multiple oral health conditions can present a challenge for the clinician when advising patients on home care choices. Many patients who need protection from dietary erosion also need help with plaque control. Products that are effective at helping to prevent both erosion and plaque-related conditions are a preferable solution. The research presented in this article was undertaken to evaluate the plaque inhibition properties of two dentifrices, which employ distinct technological routes to the provision of erosion protection. An established dentifrice (Sensodyne® ProNamel™; GlaxoSmithKline, Istanbul, Turkey) marketed under a claim to help prevent dental erosion was tested against a newly introduced dentifrice (blend-a-med* Pro Expert dentifrice; Procter & Gamble, Gross Gerau, Germany), also claiming an erosion-preventive benefit, for their ability to inhibit plaque formation *in vivo*.

## Materials and methods

**Fig. 1:**
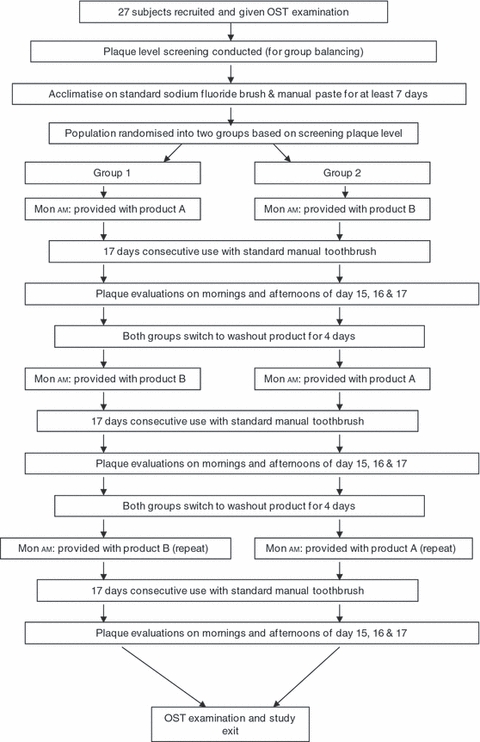
Study design.

This study used a two-treatment, three-period, randomized, double-blind, crossover design, which has been published previously (18, 19). In this design, the third period is a repeat of the treatment used in the second period, a strategy employed to control for potential carryover effects and improve statistical power. Each treatment period was for 17 days, with a 4-day washout between periods. Plaque evaluations were conducted on days 15, 16 and 17 of each treatment period early in the morning, before toothbrushing (‘am prebrushing’). In addition, on the same days, plaque evaluations were conducted immediately after early morning toothbrushing for 40 s (‘am post-brushing’) and mid-afternoon (pm). All plaque evaluations were performed using a standardized digital plaque image analysis (DPIA) method (20) (Fig. 1).

## Treatments

1. 1450 ppm NaF formulation with SnCl_2_ as key excipient. (blend-a-med* Pro Expert dentifrice; Procter & Gamble), referred to as SnCl_2_/NaF.
2. A 1450 ppm NaF dentifrice with potassium nitrate widely marketed across Europe (Sensodyne® ProNamel™; GlaxoSmithKline), referred to as NaF.

### Study design

After recruitment, all subjects underwent an oral soft tissue examination, provided a medical history and were checked against inclusion/exclusion criteria. To be eligible, subjects needed to be in good oral and general health, not have a sensitivity/allergy to dyes (especially fluorescein) and agree to refrain from use of non-study oral health products for the duration of the trial. Subjects were excluded if they were using medication (e.g. antibiotics), were pregnant or nursing, were in poor dental health, or had a dental appliance, which would interfere with the DPIA procedure. They were also excluded if they had any dental treatments (including a dental prophylaxis) carried out within 2 weeks of the start of the trial. Before the study commenced, all subjects signed the study informed consent. The application of the DPIA methodology for dentifrice research had been previously reviewed and approved by the Institutional Ethics Review Committee.

Prior to the treatment phase of the study, subjects used a standard fluoride toothpaste and manual toothbrush (Crest® Decay Prevention 1450 ppm NaF and Oral-B® P35 Indicator manual toothbrush; Procter & Gamble, Gross Gerau, Germany) twice per day for at least 7 days, to wash out any treatment effects from products used in their normal oral hygiene regimen. This standard product combination was also used during the 4-day washout between treatment periods. A balanced treatment assignment was achieved by randomizing subjects to sequences (ABB or BAA) in approximately equal numbers within each strata based on high, medium, or low pre-acclimation plaque scores, ensuring that each sequence group had a range of plaque growth rates. This was performed using recently collected data during a period when each subject was using the standard product combination.

On Monday morning of the first period, subjects were provided with a randomly assigned treatment toothbrush/toothpaste in blinded packaging (white tubes) and usage instructions. Subjects were instructed to brush twice per day as they normally would. On the Monday, Tuesday and Wednesday evenings of each week during a treatment period, subjects only brushed their lingual surfaces, while ensuring the toothpaste slurry was swished around the whole oral cavity. This previously reported design enables the chemical inhibition effects of antimicrobial pastes to be clearly demonstrated on facial surfaces (i.e. the imaged surfaces when DPIA is used) separate from the plaque effects of toothbrushing. This brushing procedure maintains a largely representative delivery of the toothpaste.

### Plaque evaluation method

Anterior facial plaque coverage was measured using DPIA. The use of DPIA for comparative product performance for plaque inhibition via hard tissue plaque coverage measurement has been reported in the literature on a number of occasions (21, 22). The imaging system used has been previously fully described by Sagel and Gerlach (23) with the reapplication for plaque analysis subsequently described by Bellamy and colleagues (20).

All subjects had am prebrushing plaque evaluated between 7:30 am and 9:30 am on evaluation days (at least 8 h after brushing the previous evening). On these mornings, subjects were required not to brush their teeth, eat, drink (except water) or smoke prior to evaluation. Five millilitres of a 1240 ppm solution of fluorescein (pH 6.0) was used to disclose the plaque. After a 1-min rinse with the fluorescein, three phosphate buffer rinses (pH 6.0) were used to stabilize mouth pH and wash away unattached dye after which immediate DPIA imaging of the facial surfaces of the front 12 teeth (canine to canine, maxillary and mandibular) was conducted. Subjects then brushed for 40 s with the test product and a post-brushing image was taken following the same disclosing and imaging procedure. Prior to the pm measure, which was taken 5–6 h after the morning brushing, subjects were required to refrain from eating and drinking for 30 min before their imaging appointment, to avoid food debris being present in the oral cavity. The same procedures were followed as with the previous two plaque assessments.

An image analysis algorithm was used to classify pixels (22) in a defined region of interest (the 12 teeth facial anterior surfaces) into one of four categories: teeth, gums, plaque or background. The percentage of the anterior facial surfaces covered with plaque was then calculated using the equation: 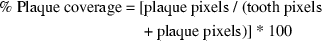


All the computer analysis output was checked by an expert in image analysis blinded to treatment for consistency and accuracy. Images that were not well classified, or of poor quality, were excluded from study results.

### Statistical methods

Percentage plaque area coverage measurements from each of 3 days were averaged separately for each subject, period and time point (am prebrush, am post-brush and pm). For each time point, analysis of variance for the crossover design (general linear mixed model) was used to compare the per cent plaque area coverage between treatments using period and treatment dentifrice as fixed effects and subject as a random effect. The carryover effect was tested for each time point, was not statistically significant (*P* > 0.31) and was subsequently removed from each statistical model. All statistical comparisons were two-sided using a 0.05 significance level.

## Results

Twenty-eight subjects were enrolled in the study; one subject was not eligible and was dropped from the study due to atypical tooth characteristics. Twenty-seven subjects were eligible for the research and completed the study, providing evaluable data at all plaque measurement periods with one exception (one subject missed Period 3). No adverse events were recorded by the investigator and no product use discomfort was reported by the subjects. All images were considered to be of sufficient quality to be included in the analysis, and no images needed to be excluded during analysis due to poor classification by the computer algorithm. Subjects ranged in age from 25 to 55 years with a mean of 34.5 years and 15 subjects were women (55.5%).

Plaque area coverage at the end of the treatment period when subjects had been using the SnCl_2_/NaF dentifrice was significantly lower (*P* < 0.0001) than when subjects had been using the NaF dentifrice. This was true for all the three time points: am prebrushing, am post-brushing and pm.Table 1 shows the mean plaque coverage at each time point and the comparative difference in plaque between the products.

**Table 1:** **Treatment comparisons for plaque area coverage (%)**

Timepoint/Treatment	Mean (SE)	% Reduction versus NaF	Two-sided *P*-value
am prebrush			
NaF	15.52 (1.10)	26.0	<0.0001
SnCl_2_/NaF	11.49 (1.11)		
am post-brush			
NaF	6.39 (0.81)	27.9	0.0001
SnCl_2_/NaF	4.61 (0.81)		
pm			
NaF	11.92 (1.08)	25.7	<0.0001
SnCl_2_/NaF	8.86 (1.08)		

Overnight plaque growth (i.e. am prebrushing) when subjects were using the SnCl_2_/NaF dentifrice was on average 26.0% less than when subjects were using the NaF dentifrice (*P* < 0.0001). A 40-s toothbrushing with either treatment reduced plaque coverage by a similar proportion (59.9% for the SnCl_2_/NaF dentifrice and 58.8% for the NaF dentifrice). However, this meant when subjects were using the SnCl_2_/NaF treatment, plaque coverage immediately after brushing was significantly lower than when subjects were using the NaF treatment (27.9% less plaque with SnCl_2_/NaF, *P* = 0.0001) as they had less plaque regrowth prior to brushing.

After a period of daytime plaque regrowth, subjects who had used the SnCl_2_/NaF dentifrice early in the morning had 25.7% less plaque coverage than subjects who were using the NaF dentifrice (*P* < 0.0001).

## Discussion

This study found that two distinctly different sodium fluoride dentifrice products developed to protect against dietary erosion differed in their ability to prevent plaque formation. The SnCl_2_/NaF dentifrice produced 25–26% lower plaque levels compared with the NaF dentifrice overnight (at least 8 h) and throughout the day (5–6 h). These differences were highly significant (*P*-values <0.0001). In addition, immediately after tooth brushing, subjects’ plaque coverage was 27.9% lower when they brushed with SnCl_2_/NaF compared with the NaF treatment.

The plaque inhibition benefit of the SnCl_2_/NaF toothpaste is attributed to the bioavailability of stannous ions in the presence of fluoride ions (24). Stannous fluoride has been shown to be an effective antiplaque agent in numerous published reports (20–22, 25, 26). A recent study using a virtually identical design compared a 0.454% stannous fluoride dentifrice with sodium hexametaphosphate as a key excipient (SnF_2_) to the same NaF dentifrice tested here (19). When subjects used the SnF_2_ dentifrice, 23.0% less plaque coverage was measured after overnight regrowth, and 22.6% less plaque coverage during the daytime compared with the NaF dentifrice, results notably similar to those reported in this study. Comparison of the SnCl_2_/NaF data reported in this article with other studies suggests plaque inhibition efficacy from the SnCl_2_/NaF dentifrice to be in the same range as this extensively tested stannous fluoride/sodium hexametaphosphate positive control (21, 22). Moreover, results for the NaF dentifrice in this trial are consistent with limited reports in the literature, which do not demonstrate efficacy for this active combination as a plaque control agent (19). Bellamy and colleagues previously reported it to be significantly inferior to a positive control dentifrice for overnight and daytime plaque inhibition (19).

While both dentifrices are marketed for their anti-erosion properties, the additional antiplaque properties demonstrated by the SnCl_2_/NaF dentifrice should be considered by dental professionals when developing home care treatment plans. Many patients at risk of dietary dental erosion also struggle with achieving optimal plaque control. It would be most efficient to address both conditions with a product that is part of a typical daily dental care routine. The twice-daily habit of toothbrushing with the SnCl_2_/NaF dentifrice presents such an opportunity

## Conclusion

Clinicians seeking to recommend a daily use dentifrice for patients susceptible to dental erosion do not need to compromise on antiplaque performance. This study showed the SnCl_2_/NaF dentifrice to be significantly more effective than a NaF toothpaste at inhibiting plaque growth both overnight and during the day in a randomized, double-blind, crossover clinical study.

## Footnotes

* Also branded Oral-B®, Crest® and Ipana®, depending on the local country brand name.
